# There are no randomized controlled trials that support the United States Preventive Services Task Force guideline on screening for depression in primary care: a systematic review

**DOI:** 10.1186/1741-7015-12-13

**Published:** 2014-01-28

**Authors:** Brett D Thombs, Roy C Ziegelstein, Michelle Roseman, Lorie A Kloda, John PA Ioannidis

**Affiliations:** 1 Department of Psychiatry, McGill University, Montréal, Québec, Canada; 2 Department of Epidemiology, Biostatistics and Occupational Health, McGill University, Montréal, Québec, Canada; 3 Department of Medicine, McGill University, Montréal, Québec, Canada; 4 Department of Psychology, McGill University, Montréal, Québec, Canada; 5 Department of Educational and Counselling Psychology, McGill University, Montréal, Québec, Canada; 6 School of Nursing, McGill University, Montréal, Québec, Canada; 7 Lady Davis Institute for Medical Research, Jewish General Hospital, Montréal, Québec, Canada; 8 Department of Medicine, Johns Hopkins University School of Medicine, Baltimore, USA; 9 Library, McGill University, Montréal, QC, Canada; 10 Stanford Prevention Research Center, Department of Medicine, Stanford University School of Humanities and Sciences, Stanford, CA, USA; 11 Department of Health Research and Policy, Stanford School of Medicine, Stanford University School of Humanities and Sciences, Stanford, CA, USA; 12 Department of Statistics, Stanford University School of Humanities and Sciences, Stanford, CA, USA

**Keywords:** Depression, Primary care, Screening, Systematic review

## Abstract

**Background:**

The United States Preventive Services Task Force (USPSTF) recommends screening adults for depression in primary care settings when staff-assisted depression management programs are available. This recommendation, however, is based on evidence from depression management programs conducted with patients already identified as depressed, even though screening is intended to identify depressed patients not already recognized or treated. The objective of this systematic review was to evaluate whether there is evidence from randomized controlled trials (RCTs) that depression screening benefits patients in primary care, using an explicit definition of screening.

**Methods:**

We re-evaluated RCTs included in the 2009 USPSTF evidence review on depression screening, including only trials that compared depression outcomes between screened and non-screened patients and met the following three criteria: determined patient eligibility and randomized prior to screening; excluded patients already diagnosed with a recent episode of depression or already being treated for depression; and provided the same level of depression treatment services to patients identified as depressed in the screening and non-screening trial arms. We also reviewed studies included in a recent Cochrane systematic review, but not the USPSTF review; conducted a focused search to update the USPSTF review; and reviewed trial registries.

**Results:**

Of the nine RCTs included in the USPSTF review, four fulfilled none of three criteria for a test of depression screening, four fulfilled one of three criteria, and one fulfilled two of three criteria. There were two additional RCTs included only in the Cochrane review, and each fulfilled one of three criteria. No eligible RCTs were found via the updated review.

**Conclusions:**

The USPSTF recommendation to screen adults for depression in primary care settings when staff-assisted depression management programs are available is not supported by evidence from any RCTs that are directly relevant to the recommendation. The USPSTF should re-evaluate this recommendation.

Please see related article: http://www.biomedcentral.com/1741-7015/12/14

**Registration:**

PROSPERO (#CRD42013004276)

## Background

Screening for depression in primary care settings is controversial [1-3]. Prior to 2002, no major guidelines recommended depression screening. Then, in 2002, the United States Preventive Services Task Force (USPSTF) recommended routine depression screening when staff-assisted depression care programs are in place to ensure accurate diagnosis and effective treatment and follow-up [4]. In 2009, the USPSTF reiterated this recommendation, based on evidence from nine randomized controlled trials (RCTs) [5,6].

By contrast, a 2008 Cochrane review [7,8] reported that the effect of depression screening on depressive symptoms in five RCTs was virtually zero (standardized mean difference = -0.02, 95% confidence interval -0.25 to 0.20) [7]. Consistent with this, in 2010, the UK National Institute for Health and Care Excellence recommended that clinicians be alert to possible depression, but not screen [9]. In 2013, the Canadian Task Force on Preventive Health Care similarly recommended against routine depression screening [10].

Existing systematic reviews on depression screening have been criticized for not defining the characteristics of depression screening trials [11,12]. Depression screening involves the use of depression symptom questionnaires to identify patients who may have depression but have not sought treatment and have not otherwise been recognized as depressed by healthcare providers. For screening to benefit patients, patients must agree to be screened, the screening test must accurately identify a significant number of previously unrecognized patients, and clinicians must engage these patients in treatment and obtain sufficiently positive results to justify costs and potential harms [3].

A trial of depression screening must be able to separate the effect of screening from the effect of providing additional treatment resources not otherwise available. In addition to screening, depression symptom questionnaires may be used for tracking symptom severity or detecting relapse among patients with already-recognized depression. However, in a trial, only patients not already under care for depression prior to the trial should be included in assessing the effect of screening, since screening is done to identify previously unrecognized cases. Thus, for a trial to test the effects of depression screening on depression outcomes, at least three key criteria must be fulfilled. The trial must:

1. determine patient eligibility and randomize patients prior to screening;

2. exclude patients already diagnosed with a recent episode of depression or being treated for depression at the time of trial enrollment;

3. provide similar depression management options to patients with depression in the screening arm of the trial and patients in the non-screening arm identified as depressed via other mechanisms, such as patient report or unaided physician diagnosis.

The 2009 USPSTF systematic review [5,6] did not explicitly define characteristics of a screening trial. The 2008 Cochrane review [7,8] excluded trials where depression care was substantially enhanced for patients in the intervention group only, but did not require randomization prior to screening or address the inclusion of already-treated patients.

The objective of the present systematic review was to determine whether the USPSTF depression screening guideline is supported by evidence that depression screening improves depression outcomes in primary care. To do this, we re-evaluated the nine RCTs included in the 2009 USPSTF systematic review on depression screening [5,6] to determine if they fulfilled the three key criteria necessary for a test of depression screening. In addition, we reviewed trials included in the 2008 Cochrane review [7,8] and conducted a focused search to determine if any depression screening trials have been conducted since those reviews.

## Methods

Methods for this systematic review were registered in the PROSPERO prospective register of systematic reviews (#CRD42013004276).

### Identification of eligible RCTs from the 2009 USPSTF and Cochrane systematic reviews

We evaluated nine RCTs from the USPSTF 2009 review and two additional RCTs that were included in one or both published versions of a Cochrane review [7,8] but not the USPSTF review. Eligible RCTs had to use a depression screening tool with a defined cut-off score to make decisions regarding further assessment or treatment of depression. In addition, patient eligibility and randomization had to occur prior to administering the screening test; patients with a recent diagnosis of depression and patients being treated for depression close to the time of trial enrollment had to have been excluded from the trial; and similar depression management resources had to have been available to patients identified as depressed in both trial arms. We included RCTs that compared depression symptom outcomes or, if not available, number of cases post-screening, but not RCTs that only reported rates of depression recognition or treatment. This is because recommendations for screening should be based on evidence of improved outcomes. Increased treatment without improved depression outcomes would expose patients to costs and potential harms but not benefit [3].

Two investigators independently reviewed full-text publications of RCTs from the USPSTF and Cochrane reviews with any disagreements resolved by consensus.

### Updated search

We searched for RCTs from any country that met eligibility criteria. The focused database search was designed based on a surveillance technique found to be the most effective method for finding new evidence to update systematic reviews in a comparison of several different methods [13]. It involved using a combination of two separate search strategies. The first was a subject search in the MEDLINE database using relevant Medical Subject Headings and text words. The second search was a ‘related citations’ search in PubMed based on the three most recent and three largest trials included in either the USPSTF or Cochrane reviews, only including studies that were described in the original publications as related to screening. Both searches were limited to RCTs by using a validated methodological hedge. The search was peer-reviewed prior to implementation on 24 April 2013, using the Ovid SP interface for MEDLINE (search 1) and the National Library of Medicine interface, PubMed (search 2). See Additional file 1.

We also searched the ClinicalTrials.gov trial registry (‘depression AND screen*’ in any field, where * retrieves terms with zero to more characters) and the World Health Organization International Clinical Trials Registry Platform (‘depression AND screen*’ in the ‘title’ field) from inception to 30 April 2013. The World Health Organization registry platform is a central database that provides access to many different clinical trial registries from around the world.

The updated review was similarly done independently by two investigators.

### Data presentation and synthesis

Since we did not identify any trials that met all three criteria necessary for tests of depression screening, synthesis of outcome data on the effect of depression screening was not possible. Instead, we reported the results of our evaluation to determine if RCTs from the USPSTF and Cochrane reviews or the updated search fulfilled the three criteria. We did not conduct an assessment of trial quality or risk of bias because no trials met criteria to be considered a test of depression screening. Two investigators independently extracted data with any disagreement resolved by consensus.

## Results

### Re-assessment of randomized controlled trials from 2009 USPSTF and Cochrane systematic reviews

As shown in Table 1, there were five RCTs [14-18] included in only the USPSTF review, four [19-22] included in both the USPSTF and Cochrane reviews, and two [23,24] included in at least one version of the Cochrane review, as well as a 2002 version of the USPSTF review [25] but not the 2009 USPSTF review. Of the 11 RCTs included in either the 2009 USPSTF or Cochrane reviews, one [20] fulfilled two of the three key criteria for a depression screening trial, six [16,17,21-24] fulfilled one, and four [14,15,18,19] did not fulfill any. Of the 11 RCTs, only two [20,21] determined trial eligibility and randomized prior to screening; only two [16,17] excluded already diagnosed and treated patients; and only four [20,22-24] provided similar depression management options to patients in both trial arms.

**Table 1:** Characteristics of randomized controlled trials in 2009 United States Preventive Services Task Force and Cochrane systematic reviews

**First author, year, country**	**Included in 2009 USPSTF, Cochrane, or both reviews or updated search**	**Number of patients randomized**	**Eligibility and randomization**	**Determined eligibility and randomized of patients analyzed prior to screening?**	**Diagnostic/treatment status**	**Excluded already diagnosed and already treated patients?**	**Depression management**	**Similar depression management options for screened and unscreened trial arms?**
Callahan 1994, US [19]	Both	175	Patients with CES-D ≥ 16 and HAMD ≥ 15 eligible and randomized to enhanced depression care versus usual care.	No	21% of enrolled patients already diagnosed and 12% already on antidepressant (overlap not specified).	No	Intervention arm: enhanced depression care.	No
							Control arm: usual care.	
Dowrick 1995, UK [23,26]	Cochrane + 2002 USPSTF^a^	116	Patients with BDI ≥14 eligible and randomized to have their BDI scores and diagnostic interpretation disclosed to their physician or not disclosed.	No	Patients excluded if their physicians believed they were currently depressed, but already diagnosed and already treated patients not necessarily excluded.^b^	No	Both groups received usual care.	Yes
Lewis 1996, UK [24]	Cochrane + 2002 USPSTF^c^	454^d^	Patients with GHQ-12 ≥2 eligible and randomized to have their GHQ-12 scores placed in their physician’s notes or not disclosed.	No	Existing depression diagnosis or treatment not in exclusion criteria. No information on depression diagnosis or treatment at time of enrollment provided.	No	Both groups received usual care.	Yes
Williams 1999, US [20]	Both	969^e^	Patients randomized to screening with single mood question, with the CES-D, or to usual care. Depression outcomes only assessed for 97 patients with major depression at baseline and a sample of 119 other patients.	Yes^e^	Only 11 of 41 diagnoses of depression post-screening were new diagnoses (27%). Patients classified as new diagnoses if no evidence of diagnosis in chart and patient reported that not diagnosed or treated in last 2 years.^f^	No	Both groups received usual care.	Yes
Wells 2000, US [14,27]	USPSTF	1356	Patients with probable depressive disorder eligible and randomized to enhanced depression care versus usual care.	No	In 6 months prior to trial, 48% of patients discussed emotional issues at medical visit; 29% had specialty mental health visit; 44% getting appropriate mental health care.	No	Intervention arm: enhanced depression care.	No
							Control arm: usual care.	
Whooley 2000, US [21]	Both	2346^g^	Patients randomized to screening with GDS and seven educational sessions versus usual care. Only 331 patients with GDS ≥6 at baseline included in depression outcome analysis.	Yes^g^	In 12 months prior to trial, 20% of patients in outcome analysis prescribed antidepressant medication.	No	Intervention arm: patients offered six weekly educational sessions on depression plus one booster session.	No
							Control arm: usual care.	
Rost 2001, US [15,28,29]	USPSTF	479	Patients with five or more symptoms of current MDD eligible. Practices randomized to enhanced depression care versus usual care.	No	In 6 months prior to trial, 44% of patients were prescribed antidepressant medication or had a specialty mental health care visit.	No	Intervention arm: enhanced depression care.	No
							Control arm: usual care.	
Jarjoura 2004, US [16]	USPSTF	61	Patients positive for depression on PRIME-MD eligible and randomized to nurse-supported depression management and referral program versus usual care.	No	Patients receiving intervention for mental health problems or seeking help for depression or other emotional problems excluded.	Yes	Intervention arm: nurse-supported depression management and referral program.	No
							Control arm: usual care.	
Bergus 2005, US [22]	Both	51	Patients with low mood or anhedonia in last two weeks based on PHQ-9 eligible and randomized to have their PHQ-9 scores disclosed to their physician or not disclosed.	No	38% of enrolled patients on medication for depression or anxiety at time of enrollment and 60% had history of depression treatment.	No	Both groups received usual care.	Yes
Bosmans 2006, The Netherlands [17]	USPSTF	145	Patients with GDS ≥5 and positive for depression on PRIME-MD eligible and randomized to enhanced depression care versus usual care.	No	Patients using antidepressants at time of trial enrollment excluded.	Yes^h^	Intervention arm: enhanced depression care.	No
							Control arm: usual care.	
Rubenstein 2007, US [18]	USPSTF	792	Patients were randomized to practices in a Veterans Administration healthcare system that provided enhanced geriatric care versus usual care. Patients positive on ≥4 of 10 GPSS items related to falls/balance, urinary incontinence, depression, memory loss, pain, weight loss, polypharmacy and general health were eligible.^i^	No^g^	Existing depression diagnosis or treatment not in exclusion criteria. No information on depression diagnosis or treatment at time of enrollment provided.	No	Intervention arm: enhanced geriatric care.	No
							Control arm: usual care.	
Yeung 2010, US [30]	Updated Search	100	Patients with confirmed MDD randomized to enhanced depression care versus usual care.	No	Patients already receiving treatment for MDD were excluded.	Yes	Intervention arm: enhanced depression care.	No
							Control arm: usual care.	
Yawn 2012, US [31]	Updated Search	2343^j^	Primary care practices were randomized to a complex depression care intervention, including screening with EPSD and PHQ-9, versus usual care. Women 5 to 12 weeks postpartum were eligible. Only 408 patients with positive depression screen at baseline included in depression outcome analysis.	Yes^j^	Existing depression diagnosis or treatment not in exclusion criteria. No information on depression diagnosis or treatment at time of enrollment provided.	No	Intervention arm: enhanced depression care.	No
							Control arm: usual care.	
Romera 2013, Spain [32]	Updated Search	3737	Primary care physician practices were randomized to training on screening with 2 questions and usual depression care versus usual care. After 6 months, 3737 patients randomly selected for depression assessment.	Yes	Existing depression diagnosis or treatment not in exclusion criteria. No information on depression diagnosis or treatment at time of enrollment provided.	No	Both groups received usual care.	Yes

^a^Dowrick *et al*. trial was included in the 2002 USPSTF review, but excluded from the 2009 USPSTF review with note, ‘Does not report included outcomes’. The exclusion note referenced only one of the published trial reports [23], which did not provide depression outcomes, but did not cite another published report [26] that did provide depression outcomes. ^b^Based on published articles and clarification provided by corresponding author. ^c^Lewis *et al*. trial was included in the 2002 USPSTF review, but excluded from the 2009 USPSTF review with note, ‘Missing both depression-specific screener and depression-specific outcomes’. The GHQ-12 appears to have been considered a depression screening and depression outcome in the 2002 review, but not in the 2009 review. ^d^The trial included three arms with 227 in each arm. One arm, however, included screening with a short questionnaire and a 15- to 45-minute computerized diagnostic interview for depression. Consistent with the Cochrane review [7] and because the diagnostic interview is not screening, we evaluated only two trial arms. ^e^Eligibility was determined and randomization occurred pre-screening. However, only 216 of 969 patients randomized (23%) were assessed for depression outcomes. ^f^Based on published article and clarification provided by corresponding author. ^g^Eligibility was determined and randomization occurred pre-screening. However, only 331 of 2,346 patients randomized (14%) were include in depression outcome analysis. ^h^Patients receiving other forms of depression treatment were not excluded, but the number of patients receiving other forms of treatment was not reported. ^i^Screening was done based on many problems faced by elderly patients. Only 46% of patients in the trial screened positive on a single item related to depression. ^j^Eligibility was determined and randomization occurred pre-screening. However, only 408 of 2,343 patients randomized (17%) were assessed for depression outcomes. BDI, Beck Depression Inventory; CES-D, Center for Epidemiological Studies Depression Scale; GDS, Geriatric Depression Scale; GHQ-12, General Health Questionnaire, 12-item version; GPSS, Geriatric Postal Screening Survey; HAMD, 21-item Hamilton Depression Rating Scale; MDD, major depressive disorder; PHQ-9; 9-item Patient Health Questionnaire; PRIME-MD, Primary Care Evaluation of Mental Disorders; USPSTF, United States Preventive Services Task Force.

Of the five RCTs [14-18] that were included in the USPSTF review but not the Cochrane review, four [14-17] were trials of complex depression care quality improvement programs and required a positive score on a depression screening tool plus a diagnosis of depression for enrolment. The other RCT [18], also a complex management intervention, was excluded from the Cochrane review because it was not specific to depression. For eligibility, elderly patients were required to have four of ten problems indicative of a potentially poor general prognosis, but not necessarily depression.

### Results from the updated search

**Figure 1:**
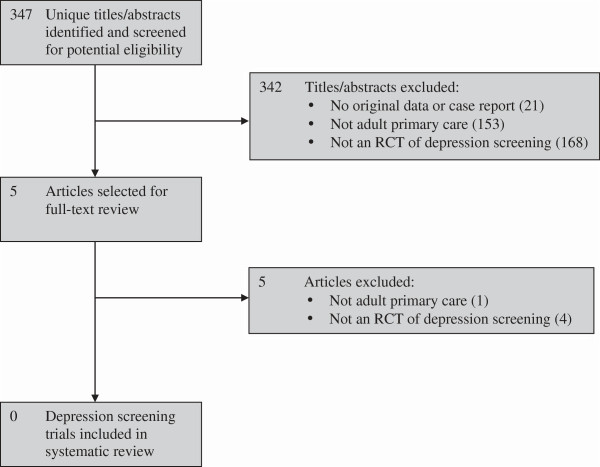
**PRISMA flow diagram of study selection process for updated search.** RCT, randomized controlled trial.

The trial registration search included 405 unique trial registrations, but none described RCTs that met eligibility criteria. The database search identified 347 unique citations, of which 342 were excluded after title and abstract review and five after full-text review (Figure 1). Of the five studies that underwent full-text review, two studies were clearly not relevant, and three RCTs [30-32] were similar in design to trials included in the USPSTF or Cochrane reviews and met at least one criterion (see Table 1). Of these three RCTs, one [30] met one, and two [31,32] met two of the three criteria.

### Randomized controlled trials that randomized patients prior to screening

Among RCTs included in the USPSTF or Cochrane reviews or identified in the updated search, only four [20,21,31,32] determined eligibility and randomized patients prior to screening. Among those, one [31] provided enhanced depression care to only patients in the screening arm, which did not allow an assessment of the effect of screening. Another [21] randomized patients to physician notification of positive screens versus usual care. Intervention arm patients with positive depression screens were offered six weekly depression education sessions, although only 12% of eligible patients attended. Outcome data were analyzed from only the 14% of patients in the trial with positive screening scores at baseline, including patients on antidepressants pre-trial. There were no significant differences in the mean change of depression symptoms between groups (-2.4 versus -2.1 points on Geriatric Depression Scale, *P* = 0.50).

Two trials [20,32] met two of the three criteria, but included already diagnosed or treated patients. One [20] analyzed data from only 23% of patients randomized, including patients determined to have major depression at baseline plus a small sample of patients without major depression at baseline. Only 27% of depression diagnoses post-screening were new diagnoses, and the mean reduction in the number of depressive symptoms did not differ between the two groups (1.6 versus 1.5 symptoms, *P* = 0.21). The other [32] was a cluster RCT in which primary care practices were randomized to screen versus provide usual care to patients at high risk of depression due to a history of depression, unexplained somatic symptoms, psychological comorbidities, drug abuse or chronic pain. The number of patients already treated pre-trial was not reported. Rates of depression post-screening were similar in the screening (15.0%) and usual care (15.8%) trial arms.

## Discussion

The main finding of this systematic review was that no RCTs have compared depression outcomes between patients randomized to be screened versus not screened for depression in trials that met the necessary criteria: determined eligibility and randomized patients prior to screening; excluded patients already known to have depression or already being treated for depression; and provided similar depression management options to patients identified as depressed via screening or via other methods in the comparison group.

The 2009 USPSTF recommendation to screen when collaborative care depression management programs are available was based primarily on the results of three trials [14,15,18]. Two of these trials [14,15] compared complex collaborative care depression management programs to usual care among patients required to have depression to enroll in the trials. In one of the trials, 44% of enrolled patients were described as receiving appropriate mental health care in the 6 months prior to trial enrollment [14]. In the other, 44% of enrolled patients were prescribed antidepressant medication or had a specialty mental health care visit in the 6 months prior to enrolling in the trial [15]. The third trial tested whether telephone case management improved a series of geriatric outcomes (depression, cognitive impairment, urinary incontinence, falls, functional impairment) among elderly patients determined to be at risk for poor health outcomes prior to trial enrollment, most of whom did not report symptoms of depression [18]. None of these trials met any of the three criteria used in the present systematic review to characterize trials of depression screening programs.

**Figure 2:**
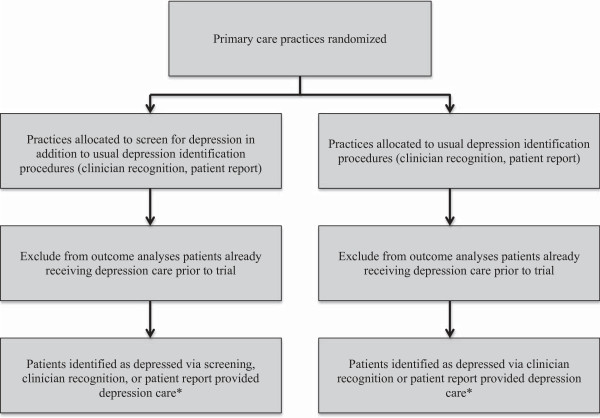
Diagram of trial to test depression screening in the context of collaborative depression care.

The trials that were used to support the USPSTF recommendation provided evidence that collaborative care programs improve outcomes compared to usual care for patients already identified as depressed. They did not, however, address the question of whether screening improves outcomes for patients who would not otherwise be identified as depressed in the context of these programs. To address this question, RCTs are needed that randomize primary care practices to screen patients for depression versus not screening them. All patients in either trial arm who are identified as depressed via screening or other mechanisms, such as unaided clinician recognition or patient report, should be provided with the same depression care to determine whether screening is linked to improved depression outcomes in the context of depression care of similar quality (Figure 2).

Without such evidence, the USPSTF should consider several factors that suggest that depression screening, even in the context of collaborative depression care, may not be straightforward or effective, and may, in fact, expose some patients to avoidable risk. These factors have been discussed in detail elsewhere [3], but include the already high rate of antidepressant use in primary care settings [33,34]; the likely overestimation in the research literature of the accuracy of depression screening tools for identifying previously unrecognized cases of depression [35]; and the limited effectiveness of antidepressant medication among patients with only mild symptoms of depression [36-41], including patients without obvious depression who would likely be identified via screening.

Although it is not clear that depression screening would improve depression outcomes, it would harm some patients [42]. Most patients treated for depression in primary care are treated with antidepressants [43], and common side effects include diarrhea, dizziness, dry mouth, fatigue, headache, nausea, sexual dysfunction, excessive sweating, tremors and weight gain [44]. Less common but potentially more serious adverse effects, particularly for patients in primary care with heart disease, may include increased risk of bleeding and unwanted effects on blood pressure and heart rate [45-49], as well as drug-drug interactions with cardiac medications [50,51]. For patients with generally low levels of depression, who are most likely to be newly identified through screening, the side effect burden and potential risk profile of antidepressants need to be carefully considered, particularly given that screening has not been shown to reduce symptoms of depression.

Screening would also consume scarce healthcare resources [52,53] that will not then be available for other activities, such as providing treatment to the large number of patients already diagnosed with depression but receiving poor-quality care. Canadian healthcare costs are generally lower than those in the US. Nonetheless, a recent population-based study from the province of Quebec found that overall healthcare costs were approximately $2,000 (CAD) higher for patients prescribed antidepressants, with a large proportion of increased costs attributed to patients without a recent history of depression or anxiety [54]. The cost of treatment, however, is only part of the cost of screening. Beyond administering depression symptom questionnaires, the cost of depression screening would include follow-up assessments to determine which patients are true positive screens and which are false positives; consultations with patients who are identified as having depression to determine management options and, if treatment is advised, the best treatment option; as well as treatment and follow-up services. The USPSTF guideline does not specify how often patients should be screened, and we do not know of any studies that have examined cumulative false positive rates from repeat screenings, but this is an important factor that needs to be considered.

Recommendations have been made for depression screening of special patient populations, including postpartum women [55] and patients with heart disease [56]. However, these recommendations are controversial [57,58] and not supported by evidence of benefit from RCTs [59-62]. In the UK, depression screening in primary care of patients with heart disease and diabetes was incentivized from 2006 to 2013. An analysis of more than a million patient records from Scottish primary care practices, however, found that almost 1,000 screens were necessary for a new depression diagnosis and almost 700 for a new antidepressant prescription [63]. In the US, at least 10 states have legislation encouraging or requiring postpartum depression screening [64]. Although no depression outcomes have been reported, a study of the first of these programs to be initiated, the New Jersey Postpartum Wellness Initiative, which has required postpartum depression screening since 2006, did not find any increase in depression treatment or follow-up care following implementation [64].

The only RCT in the present review that screened high-risk patients [32] did not find that screening reduced the presence of depression, although it is not known what proportion of patients in the trial were receiving treatment for depression pre-trial. One prospective cohort study from the Netherlands [65] documented the results of a program designed to screen and provide collaborative depression care for primary care patients with a history of mental health problems, unexplained somatic complaints, or a high level of service utilization. In that study, 1,687 patients were sent a screening questionnaire and letter from their general practitioner: 780 returned the screening questionnaire and 226 screened positive, but only 17 patients were newly diagnosed with depression and attended even one session of the offered treatment. Depression outcomes were not reported.

The present systematic review is a focused update of existing systematic reviews and did not include a complete search as in earlier reviews. Therefore, it is possible that we could have missed eligible trials. However, the updating method that we used has been validated as highly sensitive [16], and the likelihood that we have missed eligible studies that would have changed results appears to be very low.

## Conclusions

We did not find any directly relevant evidence from RCTs to support the USPSTF recommendation to screen patients for depression in primary care when staff-assisted, collaborative depression care programs are in place. This result is consistent with recent guidelines from the UK [9] and Canada [10] that concluded that routine depression screening is not supported by existing evidence. Our results differ from those of a 2008 Cochrane review [7,8], which reported that depression screening is not effective based on evidence from five trials by clarifying that there have not been any well-designed trials to directly address the question of whether depression screening may be effective, particularly in the context of collaborative care. RCTs of depression screening that are designed to directly assess whether screening of previously unidentified patients will reduce rates of depression are needed.

Over-diagnosis and over-treatment of depression are common in community and primary care settings in the US [66-68], and there is a real risk that depression screening could exacerbate this problem without contributing to better mental health. We hope that the USPSTF will re-evaluate evidence on depression screening, applying the three basic criteria that we have used in this review. Before screening for depression is recommended, there should be evidence of improved depression outcomes from well-conducted depression RCTs that are directly relevant to the question of screening.

Although our findings show that there is not enough evidence to recommend that healthcare practitioners use screening to attempt to identify patients who may have depression, depression is a disabling condition with a major impact on quality of life. Thus, clinicians should be aware of signs that depression may be present, such as low mood, loss of interest in activities, insomnia and fatigue [10]. Healthcare practitioners should be particularly vigilant among patients who may be at high risk of depression, including patients with a chronic medical condition, a past history of depression, a pattern of unexplained somatic symptoms and frequent use of medical services, or substance abuse [9,10,32,65].

## Abbreviations

RCT: Randomized controlled trial; USPSTF: United States Preventive Services Task Force.

## Competing interests

The authors declare that they have no competing interests.

## Authors’ contributions

BDT was responsible for the study concept and design; wrote the review protocol; contributed to the article review, selection and data extraction; contributed to the analysis, interpretation and presentation of data; and drafted the manuscript. RCZ contributed to the study concept and design; participated in data extraction; contributed to the analysis, interpretation and presentation of data; and provided a critical revision of the manuscript. MR contributed to the article review, selection and data extraction; contributed to the analysis, interpretation and presentation of data; and provided a critical revision of the manuscript. LAK designed and conducted the database searches and provided a critical revision of the manuscript. JPAI contributed to the study concept and design\ and to the analysis, interpretation and presentation of data, and provided a critical revision of the manuscript. All authors approved the final version of the manuscript.

## Pre-publication history

The pre-publication history for this paper can be accessed here:

http://www.biomedcentral.com/1741-7015/12/13/prepub

## Supplementary Material

Additional file 1 Search Strategy. Click here for file
