# Supplementary material for: There are no randomized controlled trials that support the United States Preventive Services Task Force guideline on screening for depression in primary care: a systematic review
Source: BMC Med. 2014 Jan 28;12:13. doi: 10.1186/1741-7015-12-13 (PMC3922694; doi:10.1186/1741-7015-12-13)
Supplement: Additional file 1 — Search Strategy. [file 1741-7015-12-13-S1.pdf]

Search Strategy

Peer reviewed by Margaret Sampson, April 18, 2013.

Search 1: Subject Search (with MeSH for population and intervention)

Population: Adults
Intervention: Screening for Major Depressive Disorder

Hedge for RCTs:
RCTs in MEDLINE: Cochrane Highly Sensitive Search Strategy for Identifying randomized trials in MEDLINE: sensitivity- and precision-maximizing version (2008 revision) (See: http://www.mrc-bsu.cam.ac.uk/cochrane/handbook/chapter_6/box_6.4.d_cochrane_hsss_2008_sensprec_ovid.htm)

MEDLINE
1. Depression/
2. Depressive Disorder/
3. Depressive Disorder, Major/
4. depress*.ti.
5. or/1-4
6. Mass Screening/
7. screening.ti.
8. 6 or 7
9. Randomized controlled trial.pt.
10. controlled clinical trial.pt.
11. randomized.ab.
12. placebo.ab.
13. clinical trials as topic.sh.
14. randomly.ab.
15. trial.ti.
16. or/9-15
17. exp animals/ not humans.sh.
18. 16 not 17
19. 5 and 8 and 18
20. limit 19 to yr="2009 -Current"
21. remove duplicates from 20

Search conducted in Ovid MEDLINE(R) In-Process & Other Non-Indexed Citations and Ovid MEDLINE (R) 1996 to April 24, 2013
[Username/password: DepressionSR/depress]
Search saved as: ScreeningUpdate April 5 2013
Results = 70

Search 2: Related Articles

3 largest trials	3 most recent trials
Whooley (2000)	Whooley (2000)
Williams (1999)	
Lewis (1996)	
	Bergus (2005)
	Jarjoura (2004)

5 included trials: Bergus (2005), Jarjoura (2004), Lewis (1996), Whooley (2000), Williams (1999)

PMIDs: 16294652 8732321 14748864 10840264 10320115

Search conducted in PubMed (NLM website) on April 24, 2013
Find related citations = 2857 records
AND "Randomized Controlled Trial"[Filter] Filters: Publication date from 2009/01/01 to 2014/12/31 = 284 records

Actual command:
(#2 AND "Randomized Controlled Trial"[Filter]) AND ("2009/01/01"[PDAT] : "2014/12/31"[PDAT])
